# 125I seed irradiation induces up-regulation of the genes associated with apoptosis and cell cycle arrest and inhibits growth of gastric cancer xenografts

**DOI:** 10.1186/1756-9966-31-61

**Published:** 2012-07-24

**Authors:** Zhen-Huan Ma, Yong Yang, Lei Zou, Kai-Yuan Luo

**Affiliations:** 1 Kunming Medical College, Kunming, Yunnan, PR of China; 2 Department of Vascular Surgery; the Second People's Hospital of Yunnan Province, Kunming, Yunnan, PR of China

## Abstract

**Background:**

Iodine 125 (125I) seed irradiation can be used as an important supplementary treatment for unresectable advanced gastric cancer. Here, we aim to comprehensively elucidate the biological effects induced by 125I seed irradiation in human gastric cancer xenograft model by using global expression and DNA methylation analyses.

**Methods:**

The 48 mice bearing NCI-N87 gastric cancer xenografts were randomly separated into 2 groups: sham seeds (O mCi) were implanted into the control group (n = 24); 125 l seeds (0.9 mCi) were implanted into the treatment group (n = 24). The mitotic index and apoptotic index were evaluated by quantitative morphometric analysis of the expression of proliferating cell nuclear antigen (PCNA) and *in situ* terminal transferase-mediated fluorescein deoxy- UTP nick end labeling (TUNEL), respectively. Global gene expression changes induced by 125I seed irradiation were analyzed by using Nimblegen Human gene expression array. DNA methylation profile in the tumors from control group was investigated with methylated DNA immunoprecipitation (MeDIP) and Nimblegen CpG promoter microarrays. The changes in the methylation status of selected genes were further investigated by using MeDIP-PCR.

**Results:**

125I seed irradiation suppresses the growth of gastric cancer xenografts in nude mice. PCNA staining and tissue TUNEL assays showed that both inhibition of cell proliferation and induction of apoptosis contribute to the 125I-induced tumor suppression in nude mouse model. Gene expression profiles revealed that the expression levels of several hundred genes, many of which are associated with apoptosis or cell cycle arrest, including BMF, MAPK8, BNIP3, RFWD3, CDKN2B and WNT9A, were upregulated following 125I seed irradiation. Furthermore, the up-regulation of some of these genes, such as BNIP3 and WNT9A, was found to be associated with irradiation-induced DNA demethylation.

**Conclusions:**

This study revealed that 125I seed irradiation could significantly induce the up-regulation of apoptosis- and cell cycle-related genes in human gastric cancer xenografts. And some of the up-regulation might be attributed to 125I-irradiation induced demethylation in gene promoter regions. Collectively, these findings provided evidence for the efficacy of this modality for the treatment of gastric cancer.

## Background

Gastric cancer is one of the most frequent cancers in the world, and almost of 50% gastric cancer death occurred in China [1-3]. Surgery offers the only realistic chance of cure; However, many of the patients present with unresectable tumors at the time of diagnosis. Even with resection, still more than 50% of patients will relapse and eventually die of their disease [4,5]. Therefore, non-surgical methods have attracted increasing attention.

In recent years, 125I implantation has been widely used to treat prostate cancer and other tumor types because of its ability to offer high precision, little trauma, strong lethality, and fewer complications [6-9]. Most recently, Wang and colleagues applied 125I implantation to treat advanced gastric cancer and found significant improvement in clinical symptoms and life quality of patients [10].

Although the 125I seed implantation have been successfully applied in clinic, its radiobiological effect and underlying molecular mechanism are far from fully understood. Recently, Zhuang and colleagues indicated that continuous low dose rate irradiation influenced the proliferation of cells *via* MAPK signal transduction. And apoptosis was the main mechanism of cell-killing effects under low dose rate 125I irradiation in CL187 cells [6]. Besides, Ma and colleagues demonstrated that 125I irradiation significantly induced cell apoptosis and inhibited DNMT1 and DNMT3b expression at 4 Gy in pancreatic cancer cells. Thus, the irradiation-induced apoptosis and DNA hypomethylation might be two key mechanisms underlying the therapeutic effect of low energy 125I seed implantation [11]. However, to date, the global molecular changes induced by 125I irradiation have not yet been fully understood. In present study, we profiled gene expression in human gastric cancer xenografts with microarrays to gain a comprehensive overview of changes induced by 125I seed irradiation.

## Methods

### Animal model

The human NCI-N87 cells (3x10 ^6^/mouse) were subcutaneously injected into right dorsal flank of each BALB/c-nu/nu nude mouse. After 1–2 weeks of implantation with tumor cells, when tumors reached ~20-30 mm 3, the animals were randomized into control and treatment groups (24 animals per group). The 125I seeds (0.9 mCi) were injected into mice in treatment group through 18-gauge needles, while ghost seed were injected into the mice in control group.The tumor size was measured using calipers and the tumor volume was estimated by the formula: tumor volume (mm3) = (L x W 2) × 1/2, where L is the length and W is the width of the tumor. Tumor volumes and body weights were monitored every 3 days over the course of treatment. The tumor weight was measured when the mouse was sacrificed. Mice were sacrificed after 28 days of treatments and tumors were removed and fixed in 10% neutral buffered formalin for histologic and immunohistochemical analyses. All animal procedures were carried out with the approval of the Animal Ethics Committee of Kunming Medical College.

### Histological analysis of tumors

Tumors were embedded in paraffin, sectioned at 5 μm, and stained with H&E (Sigma Aldrich, St. Louis, Missouri, USA). The mitotic index and apoptotic index were assessed by quantitative morphometric analysis of proliferating cell nuclear antigen (PCNA) expression and *in situ* terminal transferase-mediated fluorescein deoxy-UTP nick end labeling (TUNEL), two established markers of proliferation and apoptosis. For PCNA localization, formalin-fixed, paraffin embedded sections were incubated for 30 min with a mouse monoclonal anti-PCNA (Nova Castra Laboratories, Newcastle Upon Tyne, UK) at a 1:100 dilution. A peroxidase -conjugated antibody to mouse IgG (Abcam Inc., Cambridge, MA, USA) was applied followed by diaminobenzidine (Sigma Aldrich,St. Louis, Missouri, USA) to localize PCNA in the sections. DNA fragmentation was assessed by TUNEL, using the Apoptag Peroxidase *In situ* Apoptosis Detection Kit (Serologicals Corp., Norcross, Ga, USA). PCNA- or TUNEL-positive cells were quantified in 40 randomly selected high-power fields (x 200) of each tissue section.

### RNA extraction

Total RNA was retracted from tumors using Trizol reagent (Life Technologies Inc., Gaithersburg, Maryland, USA) according to manufacturer’s instructions. Total RNA from each sample was quantified by the NanoDrop ND-1000 (NanoDrop Technologies, Montchanin, DE, USA) and RNA integrity was assessed by standard denaturing agarose gel electrophoresis. Total RNA from one tumor from each mouse (6 tumors per group) was used for qRT-PCR analysis, whereas total RNA from tumors from four mice per group (12 tumors per group) was pooled for each microarray hybridization.

### Microarray analysis

Microarray analysis of whole-genome gene expression profiling was performed using Human 12 x 135 K Gene Expression Array (Roche Applied Science, Indianapolis, IL, USA). Double-strand cDNA (ds-cDNA) was synthesized from 5 μg of total RNA using a SuperScript ds-cDNA synthesis kit (Life Technologies Inc., Gaithersburg, Maryland, USA) in the presence of 100 pmol oligo dT primers. ds-cDNA was cleaned and labeled in accordance with the NimbleGen Gene Expression Analysis protocol (Roche Applied Science, Indianapolis, IL, USA). Microarrays were then hybridized with Cy3 labeled ds-cDNA in a hybridization chamber (Roche Applied Science, Indianapolis, IL, USA). After hybridization and washing, the slides were scanned using the Axon GenePix 4000B microarray scanner (Axon Instruments, Union City, CA, USA). Then, the data files were imported into Agilent GeneSpring Software (Agilent Technologies, Santa Clara, CA, USA) for analysis. NimbleScan software’s implementation of robust multichip average offers quantile normalization and background correction. The six gene summary files were imported into Agilent GeneSpring Software for further analysis. Genes that have values greater than or equal to lower cutoff of 50.0 in all samples were chosen for data analysis. The microarray experiment was independently repeated in triplicate for each sample group. Differentially expressed genes were identified through Fold-change and *T*-test screening. GO analysis and Pathway analysis were performed using the standard enrichment computation method.

### Real-time polymerase chain reaction (PCR)

DNase-treated total RNA extracted from each tumor sample was reverse transcribed using the Transcriptor 1st Strand cDNA Synthesis Kit (Roche Diagnostics GmbH, Mannheim, Germany). Real-time PCR was performed for quantitative analysis using SYBR green dye (TaKaRa, Tokyo, Japan) on the ABI-Prism 7900HT system (Applied Biosystems, Foster City, CA, USA) according to the protocols recommended by the manufacturer. Cycling parameters: pre-denaturation 1 min, 95°C; denaturation 15 s, 95°C; annealing 15 s, 60 °C; extension 45 s, 72°C, 40 cycles; final extension 5 min, 70°C. The fold change was calculated using the 2 -ΔΔCt method, presented as the fold-expression change in irradiated tumors relative to control tumors after normalization to the endogenous control, GAPDH. All experiments were carried out in triplicate technically. All primers are listed in Additional file 1: Table S1.

### Methyl-DNA immunoprecipitation and microarray hybridization

Genomic DNA from tumors from six mice in the control group was pooled for Methyl-DNA immunoprecipitation (MeDIP) experiment. MeDIP was performed as described previously [12]. Briefly, Genomic DNA was sonicated to produce random fragments in size of 200–600 bp. Four micrograms of fragmented DNA was used for a standard MeDIP assay as described. After denaturation at 95°C for 10 min, immunoprecipitation was performed using 10 μg monoclonal antibody against 5-methylcytidine in a final volume of 500 μL IP buffer (10 mmol/L sodium phosphate, pH 7.0), 140 mmol/L NaCl, 0.05% Triton X-100) at 4°C for 2 h. Immunoprecipitated complexes were collected with Dynabeads Protein A and M-280 sheep anti-mouse IgG (Roche Diagnostics GmbH, Mannheim, Germany) at 4°C for 12 h, washed with 1 × IP buffer for 4 times, treated with Proteinase K at 50°C for 4 h, and purified by phenol-chloroform extraction and isopropanol precipitation. Immunoprecipitated methylated DNA was labeled with Cy5 fluorophere and the input genomic DNA was labeled with Cy3 fluorophere. Labeled DNA from the enriched and the input pools was combined (1–2 μg) and hybridized to a NimbleGen HG18 CpG promoter Array (Roche Diagnostics GmbH, Mannheim, Germany), which contained all well-characterized RefSeq promoter regions [from −800 bp to +200 bp transcription start sites (TSSs)]. Array was then washed and scanned with Axon GenePix 4000B microarray scanner. After normalization, raw data was input into SignalMap software (Roche Diagnostics GmbH, Mannheim, Germany) to observe and evaluate the methylation peaks. A customized peak-finding algorithm provided by NimbleGen was applied to analyze methylation data from MeDIP-microarray as previously described. The algorithm was used to perform the modified Kolmogorov-Smirnov test on several adjacent probes using sliding windows to predict enriched regions across the array.

### MeDIP-quantitative PCR assay

A MeDIP assay, combined with qPCR, was used to evaluate quantitatively the methylation status of candidate genes in the tumors derived from the control and 125I treatment groups. MeDIP was performed as described above. Purified DNA from the immunoprecipitated DNA complexes and from input DNA was analyzed by qRT-PCR on an Applied Biosystems 7900 Real- Time PCR System. The experiment was performed in triplicate. The relative changes in the extent of gene methylation were determined by measuring the amount of detected genes in immunoprecipitated DNA after normalization to the input DNA. The primer sequences are listed in Additional file 1: Table S1.

### Statistical analysis

The results of the animal experiments and real-time PCR were analyzed using SPSS 13.0 software. (SPSS Inc., Chicago, IL, USA) All data were plotted as mean ± standard deviation. Student’s *t*-test was used to compare values between two independent groups. Differences were considered to be significance when p < 0.05.

## Results

Inhibitory effect of I125 seed irradiation on the growth of gastric cancer

**Figure 1:**
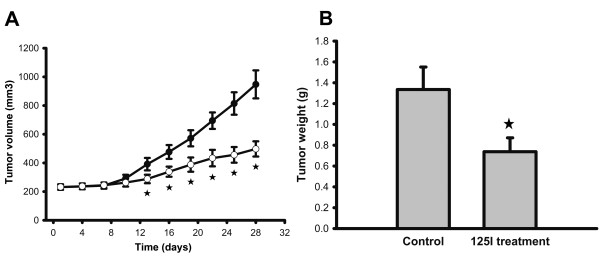
** Effect of 125I seed irradiation on the tumor volume and tumor weight.** (**A**) Tumor volumes. (**B**) Tumor weight. Data are the mean ± SD and analyzed by the Mann–Whitney *U* test (☆: P < 0.05).

The effectiveness of 125I seed irradiation to inhibit the growth of implanted NCI-N87 tumors was examined in nude mouse model. There were no significant changes in the tumor volumes for the first 10 days of the 125I seed treatment. However, after 13 days, the 125I-irradiated tumors were much smaller, and significant differences in tumor volumes were observed over time between the control and 125I treatment groups Figure 1A). At day 28, the mice were sacrificed and tumor weights were measured. Statistical difference in the tumor weight was observed between the control and treatment groups Figure 1B). All these data clearly indicated that 125I seed implantation could effectively inhibit tumor growth. Besides, the body weights of mice were not affected by the 125I irradiation and no obvious radiation-induced damage was observed in vital organs of mice (data not shown), indicating the safety of 125I seed treatment.

### Effect of 125I seed irradiation on tumor morphology of gastric cancer

**Figure 2:**
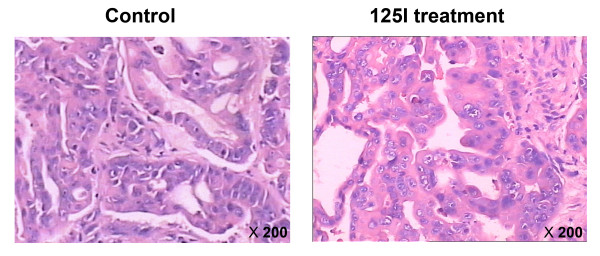
** Pathology of 125 I implanted gastric cancer.** Representative HE stained sections from the control and 125I treatment groups 28 d after 125 I seed implantation were prepared as described in the Materials and Methods section.

To investigate the effect of 125I irradiation on the histology of NCI-N87 xenografts, tumor sections taken from mice in the control and 125I treatment groups were stained with H&E. As shown in Figure 2, the histologic appearance of tumors in the control group was quite different from that in the 125I treatment group. In the control group, the cancer cells were densely arranged with large darkly-stained nuclei and obvious karyokinesis. In the treatment group, large necrotic regions were observed around the 125I seed. The cancer cells adjacent to the necrotic region were loosely arranged with condensed nuclei and reduced eosinophilic cytoplasm. These results indicated that 125I seed implantation caused growth inhibition of cancer cells in NCI-N87 xenografts.

### Effect of 125I seed irradiation on cell apoptosis and mitosis of gastric cancer

**Figure 3:**
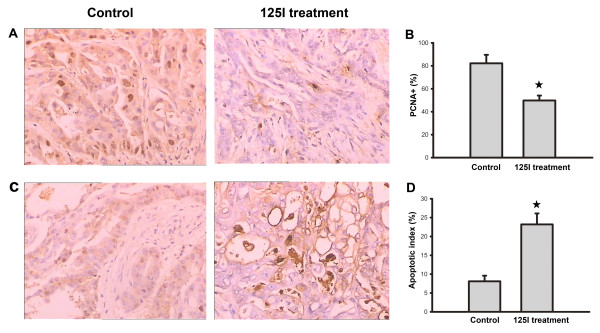
** PCNA and TUNEL analyses for tumor tissue.** (**A**) Tumor sections immunostained with an antibody against PCNA revealed that there were many strongly positive nuclei in control tumor tissues, whereas such nuclei were rare in tumor tissues of 125I treatment group. (**B**) Quantification of PCNA staining showed mitotic index of 125I-implanted tumor was much lower than that of control group (☆: P < 0.05). (**C**) Apoptosis of tumor tissues in different groups were evaluated by TUNEL assays, which showed that 125I treatment induced a significant enhancement of apoptotic cells in contrast to control group. (**D**) Quantification of TUNEL assay showed that apoptosis index of 125I-implanted tumor was much higher than that of control group (☆: P < 0.05).

To quantitatively compare the mitotic and apoptotic index of tumors treated with 125I seed irradiation, immunostainings for PCNA and TUNEL assays were performed. As shown in Figure 3A, the number of PCNA- positive cells in the 125I treatment group was obviously less than that of control group. And the mitotic index was significantly decreased in irradiated tumors as compared to the tumors in the control group Figure 3B). In contrast to the mitotic index, 125I-irradiated tumors showed increased numbers of apoptotic cells with condensed and irregularly shaped nuclei, staining positively for TUNEL Figure 3 C). the apoptotic index was significantly increased in the 125I treatment group as compared to the control group Figure 3D).

### Identification of genes induced by 125I seed irradiation

Gene expression microarrays were used to characterize the gene expression changes in NCI-N87 tumors between the 125I treatment group and control group. When the Fold Change (FC) is set > 1.3 and the p value at ≤ 0. 05, we found that 544 genes were induced by 125I seed irradiation, while 368 genes were repressed (Additional file 2: Table S2). To identify the biological processes that were induced by 125I seed irradiation, Gene Ontology (GO) functional analysis was performed. GO terms for biological processes were assigned to these differential genes and this procedure was essential to provide an overview of the effect of 125I seed implantation in NCI-N87 xenografts. According to GO functional analysis, the categories cell cycle, induction of apoptosis, cell division and growth were most significantly overrepresented among the 125-irradiation induced genes (Additional file 3: Table S3). And many of these genes are critical pro-apoptotic molecules or genes associated with cell cycle arrest, such as MAPK8, BNIP3 and CDKN2B (Table 1). Then, we employed DAVID software on the basis of the KEGG pathway map to further investigate key pathways linked to these genes. Our analysis yielded 11 pathways, including cell cycle pathway and several pathways associated apoptosis and cell cycle arrest, such as MAPK and TGF-beta signaling pathways (Additional file 4: Table S4).

**Table 1:** 125I-irradiation induced genes associated with apoptosis and cell cycle arrest

**GENE_NAME**	**DESCRIPTION**	**Fold change**	**P value**	**FDR**
**Pro-apoptotic genes**				
BNIP3	BCL2/adenovirus E1B 19 kDa interacting protein 3	2.1	0.045	0.050
MAPK8	mitogen-activated protein kinase 8	1.7	0.017	0.047
BCL2L11	BCL2-like 11 (apoptosis facilitator)	1.9	5.39E-04	0.036
AKT1	v-akt murine thymoma viral oncogene homolog 1	1.4	0.028	0.049
BMF	Bcl2 modifying factor	1.5	0.005	0.040
P2RX7	purinergic receptor P2X, ligand-gated ion channel, 7	1.4	0.004	0.040
TNFRSF10B	tumor necrosis factor receptor superfamily, member 10b	1.4	0.003	0.038
APH1A	anterior pharynx defective 1 homolog A (C. elegans)	1.4	0.010	0.039
TRAIP	TRAF interacting protein	1.4	0.032	0.046
JAK2	Janus kinase 2 (a protein tyrosine kinase)	1.6	0.011	0.045
TRIM35	tripartite motif-containing 35	1.3	0.018	0.046
ITSN1	intersectin 1 (SH3 domain protein)	1.5	0.020	0.046
TAP2	transporter 2, ATP-binding cassette, sub-family B (MDR/TAP)	1.3	0.024	0.048
ACVR1B	activin A receptor, type IB	1.6	0.009	0.046
**Genes associated with cell cycle arrest**				
CDKN2B	cyclin-dependent kinase inhibitor 2B (p15, inhibits CDK4)	1.3	0.034	0.049
RFWD3	ring finger and WD repeat domain 3	1.3	0.040	0.050
HUS1	HUS1 checkpoint homolog (S. pombe)	1.4	0.017	0.047
PMP22	peripheral myelin protein 22	1.5	0.042	0.050
CDC25C	cell division cycle 25 C	1.5	0.017	0.047
WNT9A	wingless-type MMTV integration site family, member 9A	1.6	0.048	0.050
CCNG2	cyclin G2	1.5	0.003	0.038
CDC7	CDC7 cell division cycle 7 (S. cerevisiae)	1.4	0.016	0.049
C12orf32	chromosome 12 open reading frame 32	1.5	0.002	0.033

**Figure 4:**
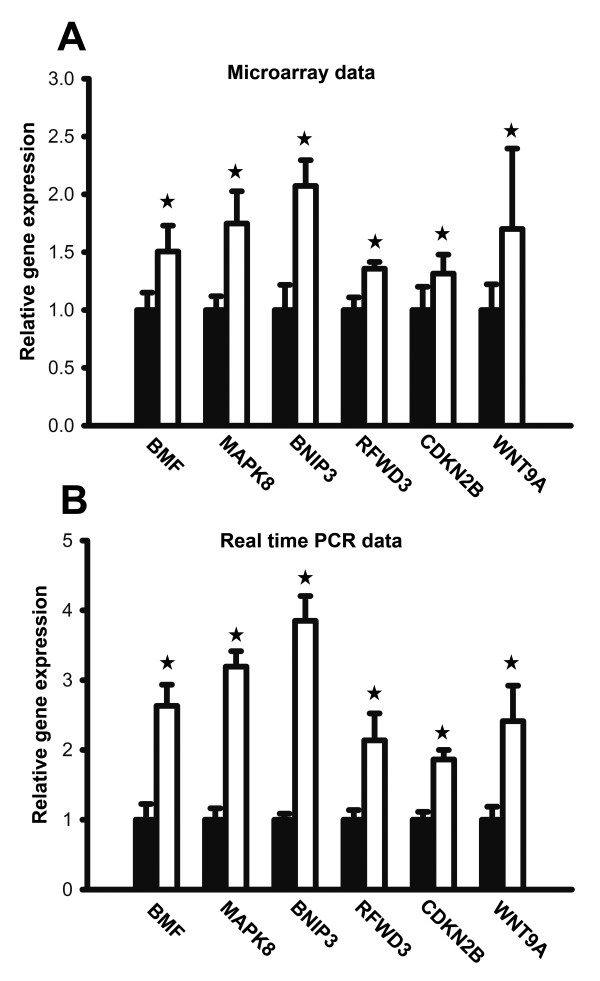
** Quantitative RT-PCR validation for differential genes in microarrays.** (**A**) Relative mRNA expression of 6 selected genes in microarrays (**B**) Validation of relative mRNA expression of selected genes with qRT-PCR. The significance of the varieties between the control group (solid bar) and 125I treatment group (hollow bar) was analyzed through student’s t-*t* test. (☆: P < 0.05).

To independently confirm the microarray results, real-time RT PCR was performed on samples from BALB/c mice that had been exposed to the same experimental conditions that were used in microarray assay. The relative expression levels of six genes—BMF, MAPK8, BNIP3, RFWD3, CDKN2B and WNT9A—were assayed in irradiated and non-irradiated tumors. There was a close correlation between microarray data and qRT-PCR data Figure 4), indicating the accuracy of our microarray data and the significant induction in the expression of selected genes following irradiation.

Collectively, these data indicated that many critical molecules and pathways associated with apoptosis and cell cycle arrest were activated by 125I seed irradiation in NCI-N87 xenografts, thereby highlighting their important roles in 125I irradiation-induced inhibition of tumor growth.

### DNA methylation analysis of 125I-irradiation induced genes

**Figure 5:**
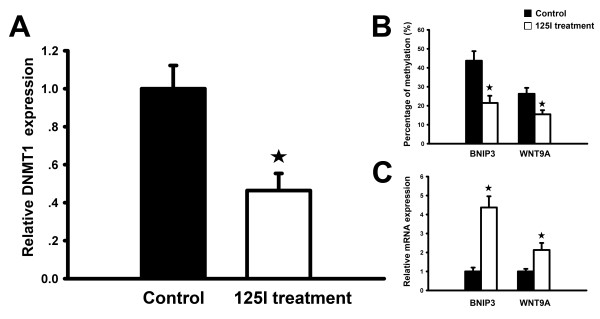
** Effects of 125I irradiation on gene methylation and mRNA expression in xenografts.** (**A**) Relative expression of DNMT1 was detected using qRT-PCR. (**B**) Effects of 125I irradiation on gene methylation of BNIP3 and WNT9A in xenografts assayed by MeDIP-PCR. BNIP3 and WNT9A in treatment group displayed lower level of methylation when compared with control group. (**C**) Relative expression of BNIP3 and WNT9A was detected using qRT-PCR. Data are expressed as the mean ± SD of 6 samples. The significance of the varieties between the control group and 125I treatment group was analyzed through student’s t-*t* test. (☆: P < 0.05).

Aberrant DNA hypermethylation is commonly associated with cancer. The Dnmt1 DNA methyltransferase is responsible for maintenance of the DNA methylation pattern. Consistent with previous study [11], significant decrease of DNMT1 expression was observed in our array data, and this result was validated *via* the real time RT-PCR Figure 5A). These data suggest that DNA demethylation might be involved in 125I-induced tumor suppression. Because promoter demethylation is associated with gene re-activating, we focused our attention on the 125I irradiation-induced genes by coupling global gene expression and methylation profiles. The genes with promoter hypermethylation in the non-irradiated tumors were indentified with MeDIP-chip analysis (Additional file 5: Table S5). Among them, we identified 20 genes whose expression was significantly upregulated in the irradiated tumors as compared to the non-irradiated tumors (Table 2). Thus, we speculated that the expression levels of these 20 genes might be modulated *via* the promoter demethylation induced by 125I irradiation. Notably, several of these genes were associated with apoptosis or cell cycle arrest, such as BNIP3, WNT9A and GSG2. To confirm our hypothesis, methylation status of these three genes was examined with MeDIP-PCR assay in the treatment and control groups. As shown, BNIP3 and WNT9A in 125I treatment group displayed lower levels of methylation status compared with control group (P < 0.05), which decreased to 50.9% and 41.0%, respectively Figure 5B). Meanwhile, the expression levels of BNIP3 and WNT9A were significantly upregulated in the treatment group Figure 5 C). These data indicated that some apoptosis- and cell cycle-related genes could be activated by the demethylation of their promoters, which were induced by 125I seed irradiation. 

**Table 2:** The irradiation-induced genes with promoter hypermethylation in the non-irradiated tumors

**GENE_NAME**	**DESCRIPTION**	**Fold change**	**Regulation**	**P-value**	**FDR**
DRD5	dopamine receptor D5	1.4	up	2.85E-04	0.03
PFN2	profilin 2	1.4	up	0.021	0.05
SKI	v-ski sarcoma viral oncogene homolog (avian)	1.6	up	0.005	0.04
WNT9A	wingless-type MMTV integration site family, member 9A	1.6	up	0.048	0.05
CXorf12	chromosome X open reading frame 12	2.0	up	0.012	0.05
BNIP3	BCL2/adenovirus E1B 19 kDa interacting protein 3	2.0	up	0.045	0.05
CHST10	carbohydrate sulfotransferase 10	2.2	up	0.010	0.05
PNMA1	paraneoplastic antigen MA1	1.3	up	0.001	0.04
C18orf55	chromosome 18 open reading frame 55	1.4	up	0.009	0.05
TRAK2	trafficking protein, kinesin binding 2	1.3	up	0.047	0.05
LRRC49	leucine rich repeat containing 49	1.5	up	0.041	0.05
EPB41L4B	erythrocyte membrane protein band 4.1 like 4B	1.4	up	0.027	0.05
USP31	ubiquitin specific peptidase 31	1.5	up	0.021	0.05
GSG2	germ cell associated 2 (haspin)	1.6	up	0.035	0.05
ATAD1	ATPase family, AAA domain containing 1	1.3	up	0.006	0.04
MGC16385	hypothetical protein MGC16385	1.4	up	0.046	0.05
TCEB3C	transcription elongation factor B polypeptide 3 C (elongin A3)	2.0	up	0.006	0.04
LONRF1	LON peptidase N-terminal domain and ring finger 1	1.4	up	0.014	0.05
SAMD11	sterile alpha motif domain containing 11	1.4	up	0.031	0.05
SLC35E2	solute carrier family 35, member E2	1.3	up	0.027	0.05

## Discussion

Several recent studies have suggested that apoptosis and cell cycle arrest may have important roles in the therapeutic effects of the continuous low-energy 125I irradiation. However, the comprehensive evidences on this topic, especially in molecular levels, still lack. In this study, microarray analysis of human gastric cancer xenografts exposed to 125I seed irradiation were performed to gain insight into the mechanisms underlying the biological effects of 125I irradiation.

N87 gastric cancer cells were implanted into the nude mice to create the xenograft animal model. The growth curves of tumors indicated that irradiation induced significant tumor growth inhibition. By observing H.E. staining slides, large numbers of apoptotic cells were observed in gastric cancer receiving 125I seeds implantation. Furthermore, the mitotic index and apoptotic index were assessed by quantitative morphometric analysis of PCNA expression and TUNEL, respectively. In our work, a declined mitotic index and increased apoptotic index were discerned in 125I treatment group compared with control group, which suggests that 125I seed irradiation can restrain tumor growth and lead to apoptosis of cancer cells.

Next, we use microarray gene expression profile analysis to study the mechanism of irradiation-mediated prevention of gastric tumors. To our knowledge, this is the first investigation to use microarray technology to study the role of 125I seed irradiation in cancer treatment. At 28 days following 125I seed irradiation, the nude mice were sacrificed and gene expression was profiled in the xenografts by using gene expression microarrays. We found that the expression levels of 544 genes were significantly induced by 125I seed irradiation. Interestingly, among the irradiation-induced genes, many are involved in cell cycle, apoptosis and cell division. The main pathways linked to these genes were further investigated by KEGG analysis and several apoptosis- or cell cycle-related pathways, such as MAPK and TGF-beta pathways, were clearly indentified. Then, the expression of 6 genes (BNIP3, MAPK8, BMF, RFWD3, CDKN2B and WNT9A), which were associated with apoptosis or cell cycle arrest, was further validated *via* real time PCR analysis Figure 3). BNIP3 (BCL2/adenovirus E1B 19 kDa interacting protein 3) is a proapoptotic member of the Bcl-2 family and its mutation and dysregulation might play a role in gastric carcinoma development [13]. Recent study revealed that BNIP3 might play a role in enhancement of radiotherapy efficiency, and its expression might have a synergistic effect on radiation treatments [14]. MAPK8 (Mitogen-activated protein kinase 8) is a member of the MAP kinase and JNK family. This gene is involved in UV radiation-induced apoptosis, which is thought to be related to the cytochrome c-mediated cell death pathway [15]. BMF (Bcl-2-modifying factor) is a Bcl-2 family member bearing only the BH3 domain and an essential inducer of apoptosis [16]. BMF contributes to enhancing effects on apoptosis after ionizing radiation [17]. RFWD3(ring finger and WD repeat domain 3) is an E3 ubiquitin ligase that positively regulates p53 levels and regulates G1 Checkpoint in Response to ionizing radiation [18]. CDKN2B (Cyclin-dependent kinase 4 inhibitor B) belongs to a family of cyclin-dependent kinase 4 inhibitors (INK41) and controls cell proliferation during the G1 phase of the cell cycle [19]. The expression of this gene was found to be dramatically induced by TGF beta, which suggested its role in the TGF beta induced growth inhibition [20]. WNT9A is a member of the WNT gene family and over-expression of t human Wnt9a induced cell-cycle arrest at G1/S boundary [21].

Consistent with previous study [11], we found significantly decreased expression level of DNMT1 in the irradiated xenografts. DNMT1 is responsible for precise duplicating and maintaining the pre-existing DNA methylation patterns after replication [22]. Therefore, it is reasonable to speculate that DNA hypomethylation induced by 125I irradiation might be associated with tumor growth inhibition. By coupling data derived from gene expression microarrays with that of MeDIP-chip, we found 39 candidate genes whose expression might be activated by 125I-induced DNA demethylation. Notably, several of the candidates are pro-apoptotic molecules or genes associated with cell cycle arrest, such as BNIP3, WNT9A and GSG2 (Serine/threonine-protein kinase haspin). The promoter demethylation of BNIP3 and WNT9A after receiving 125I irradiation was then successfully validated with MeDIP-PCR. DNA methylation of the BNIP3 promoter was mediated by DNMT1 *via* the MEK pathway [23]. Aberrant methylation of BNIP3 was also detected in 66% of primary colorectal and 49% of primary gastric cancers. Epigenetic alteration of BNIP3 is a frequent and cancer-specific event, which suggests that inactivation of BNIP3 likely plays a key role in the progression of some gastrointestinal cancers and that it may be a useful molecular target for therapy [24]. Methylation of WNT9A promoter occurs frequently in primary colon cancers and WNT9A hypermethylation in cancer points to its possible role as a tumor suppressor gene [25].

This study provides first demonstration for the global induction of apoptotic and cell cycle-related genes by 125I seed irradiation. And some of the induction may be mediated by the irradiation-induced DNA demethylation, suggesting that 125I seed irradiation affects genes associated with apoptosis and cell cycle arrest in both transcriptional and epigenetic levels. Collectively, these data provide an explanation for the tumor inhibitory effect of 125I seed implantation and emphasize the important roles of apoptosis and cell cycle arrest underlying the efficacy of this modality.

## Competing interests

The authors declare that the y have no competing interests.

## Authors’ contributions

MZH carried out animal experiment, histological analysis, molecular genetic studies, statistical analyses and drafted the manuscript. YY contributed to animal experiment and TUNEL staining. ZL participated in histological analysis and statistical analyses. LKY conceived of the study and designed the topic. All authors read and approved the final manuscript.

## Supplementary Material

Additional file 1 The sequences of PCR primers. Click here for file

Additional file 2 ** List of genes induced or repressed by 125I irradiation.** Fold change and P values are the results comparing treatment group to control group. Click here for file

Additional file 3 ** Biological processes overrepresented among the irradiation induced or repressed genes.** “Selection Counts” stands for the Count of the 125I-irradiation induced genes’ entities directly associated with the listed GO category; “Count” stands for the count of the chosen background population genes’ entities associated with the listed GO category. Click here for file

Additional file 4 The most enrichment pathways among genes related to cell cycle, apoptosis, cell division and growth by KEGG. Click here for file

Additional file 5 The genes with promoter hypermethylation in the non-irradiated tumors. Click here for file
